# Integrative analysis of metabolome and transcriptome reveals the mechanism of color formation in cassava (*Manihot esculenta* Crantz) leaves

**DOI:** 10.3389/fpls.2023.1181257

**Published:** 2023-06-09

**Authors:** Xiuqin Luo, Feifei An, Jingjing Xue, Wenli Zhu, Zhuowen Wei, Wenjun Ou, Kaimian Li, Songbi Chen, Jie Cai

**Affiliations:** Tropical Crops Genetic Resources Institute, Chinese Academy of Tropical Agricultural Sciences/Key Laboratory of Ministry of Agriculture and Rural Affairs for Germplasm Resources Conservation and Utilization of Cassava, Haikou, China

**Keywords:** cassava, transcriptomics, metabolomics, anthocyanins accumulation, VIGS

## Abstract

Cassava (*Manihot esculenta* Crantz) leaves are often used as vegetables in Africa. Anthocyanins possess antioxidant, anti-inflammatory, anti-cancer, and other biological activities. They are poor in green leaves but rich in the purple leaves of cassava. The mechanism of anthocyanin’s accumulation in cassava is poorly understood. In this study, two cassava varieties, SC9 with green leaves and Ziyehuangxin with purple leaves (PL), were selected to perform an integrative analysis using metabolomics and transcriptomics. The metabolomic analysis indicated that the most significantly differential metabolites (SDMs) belong to anthocyanins and are highly accumulated in PL. The transcriptomic analysis revealed that differentially expressed genes (DEGs) are enriched in secondary metabolites biosynthesis. The analysis of the combination of metabolomics and transcriptomics showed that metabolite changes are associated with the gene expressions in the anthocyanin biosynthesis pathway. In addition, some transcription factors (TFs) may be involved in anthocyanin biosynthesis. To further investigate the correlation between anthocyanin accumulation and color formation in cassava leaves, the virus-induced gene silencing (VIGS) system was used. VIGS-*MeANR* silenced plant showed the altered phenotypes of cassava leaves, partially from green to purple color, resulting in a significant increase of the total anthocyanin content and reduction in the expression of *MeANR*. These results provide a theoretical basis for breeding cassava varieties with anthocyanin-rich leaves.

## Introduction

Cassava (*Manihot esculenta* Crantz) is the sixth staple food crop in the world (An et al., 2019; Cai et al., 2021), which feeding more than a billion people, particularly in sub-Saharan Africa (Kumba, 2012). Cassava is an energy-producing tuberous crop with starchy root which is not only used for industrial materials but also acts as food in southern China. The leaves are also a kind of forage full of protein, flavonoids, carotenoids, and so on. In Africa, cassava leaves are usually consumed as a vegetable in daily life. Most cassava leaves are green in color; however, different cassava germplasms have different characteristics in the leaves. Some specific cassava germplasms have purple leaves, which are rich in flavonoids, especially anthocyanin. Recent studies indicated that flavonoids are detected in the leaves or storage roots of cassava (Omar et al., 2012; Xiao et al., 2021; Fu et al., 2022). However, metabolomic and genetic differences in anthocyanin biosynthesis of cassava leaves in those specific germplasms are poorly understood.

Anthocyanin is an important component of flavonoids (Chen et al., 2014), which plays several functions in plant development. Anthocyanins are involved in plant physiological processes such as attracting pollinators and seed dispersers, combatting abiotic stress, and promoting plant defense (Forkmann, 2010; Zaynab et al., 2018; Sharma et al., 2019). As water-soluble pigments contribute to the majority of the purple, blue, and red colors, anthocyanins are involved in the color formation of leaves, flowers, and fruits (Bosse et al., 2021). As a strong antioxidant, anthocyanins also have a wide range of medicinal values such as antidiabetic, anticancer, antineurodegeneration, and antineuroinflammation, as well as treating cardiovascular diseases (Bai et al., 2019; Calis et al., 2020; Park et al., 2022; Ciumărnean et al., 2020). Therefore, understanding the biosynthesis and regulation mechanism of anthocyanins is very important for breeding anthocyanin-rich cassava varieties, especially for the nutritional intake of Africans.

The biosynthesis of anthocyanins is composed of three branches and is catalyzed by a series of enzymes in the phenylpropanoid and isoflavonoid biosynthetic pathways (Quattrocchio et al., 2006; Ferreyra et al., 2012). Anthocyanin is synthesized from malonyl CoA and coumaroyl-CoA. Naringenin is produced from 1 coumaroyl CoA and 3 malonyl-CoAs, desaturated by chalcone synthase (CHS), and then isomerized by chalcone isomerase (CHI). As a core intermediate for anthocyanin biosynthesis, naringenin is catalyzed by a series of enzymes to form different types of flavonoids, such as flavonoid 3′-hydroxylase (F3′H), flavonoid 3′5′-hydroxylase (F3′5′H), and naringenin 3-dioxygenase (N3D), then catalyzed by dihydroflavonol 4-reductase (DFR), anthocyanidin synthase (ANS), and anthocyanin reductase (ANR) to form a series of derivatives. However, the anthocyanins produced in the cytoplasm are unstable and require further glycosylation modification by UDP glucosyltransferase (UGT; named BZ1 in cassava), which is ultimately transferred to vacuoles and stored together with glutathione S-transferase (GST). (Winkel-Shirley, 2001; Jaakola, 2013). Nevertheless, the biosynthesis mechanism of anthocyanins in cassava is poorly understood. There are unknown reasons why purple leaves have more anthocyanins than green leaves. Therefore, it is necessary to explore the specific mechanism of anthocyanin biosynthesis in cassava.

Ultra-Performance Liquid Chromatography-tandem/Electron Spray Ionization-Quadrupole TRAP-Mass Spectrometry/Mass Spectrometry (UPLC/ESI-Q TRAP-MS/MS) is widely used in the identification and analysis of plant metabolite and has the advantages of high sensitivity and throughput, fast separation, and wide coverage. This technology has been widely applied to analyze the metabolites in tomatoes, strawberries, and asparagus (Paolo et al., 2018; Zhu et al., 2018). In recent years, an integrative analysis of metabolomics and transcriptomics was used to analyze the correlation between metabolites and genes expression in plants (Jiang et al., 2020; Liu et al., 2020; Xu et al., 2021; Zou et al., 2021).

In this study, an integrative analysis of metabolomics and transcriptomics was performed on two cassava varieties, SC9 with green leaves and PL with purple leaves, from the National Cassava Germplasm Repository (NCGR) in China to investigate the mechanism of anthocyanin biosynthesis in cassava leaves. Significantly different metabolites (SDMs) and differentially expressed genes (DEGs) involved in anthocyanin biosynthesis were analyzed. The virus-induced gene silencing (VIGS)-induced phenotypes were also characterized. Our study provides the candidate genes and further explains the anthocyanin biosynthetic pathway in cassava. It also laid the foundation to develop functional substances and promote the cassava value chain.

## Materials and methods

### Plant material and sampling

Two cassava varieties, SC9 (Green leaves) and PL (Purple leaves), provided by the National Cassava Germplasm Repository were planted in the field (19°30’33.13”N, 109°30’19.34”E) located at Danzhou City, Hainan Province, China, in February. The young leaves of SC9 and PL were collected 4 months after planting. The leaves of SC9 and PL were frozen in liquid nitrogen and stored at -80°C for RNA and metabolite extraction. The cassava leaf sample was ground into powder (30 Hz, 1.5 min) in liquid nitrogen. For RNA extraction using the RNA extraction kit (Tiangen), 0.1g powder was weighted, and 0.05 g powder was weighted for metabolites extraction with 0.5 mL methanol/water/hydrochloric acid (500:500:1, V/V/V). The metabolite extract was vortexed for 5 min, ultrasound was performed for 5 min and was centrifuged for 3 min at 12,000 rpm. All the steps were done at a temperature of 4°C. The residue was re-extracted by repeating the above steps with the same conditions. The supernatants were collected and filtrated through a 0.22 μm membrane filter before LC-MS/MS analysis. Three biological replicates were performed for all experiments.

### Library preparation and transcriptome sequencing

RNA extraction and qualification were performed according to the instruction by An et al. (An et al., 2022). NEBNext® UltraTM RNA Library Prep Kit (New England Biolabs, USA) was used to prepare sequencing libraries by Illumina® following the instruction and index codes. The library fragments were purified with the AMPure XP system (Beckman Coulter, Beverly, USA) to select cDNA fragments ranging from 250 to 300 bp in length. Purified (AMPure XP system) PCR products and library quality were assessed on the Agilent Bioanalyzer 2100 system.

The clustering of the index-coded samples was performed on a cBot Cluster Generation System using TruSeq PE Cluster Kit v3-cBot-HS following the instructions. Then, the library preparations were sequenced on an Illumina Hiseq platform and 125 bp/150 bp paired-end reads were generated. In this study, the coding sequences (CDSs) predicted from the Phytozome (https://phytozome-next.jgi.doe.gov/) were annotated through KEGG (https://wwwkegg.jp/kegg), GO (https://geneontology.org), NR (https://www.ncbi.nlm.nih.gov/), Swiss-Prot (https://www.uniprot.org/), trEMBL (https://www.uniprot.org/), and KOG (https://www.ncbi.nlm.nih.gov/research/cog-project/) databases. PL was used as an indicator to measure transcript or gene expression levels. DESeq2 was used to identify DEGs with absolute log_2_fold change of equal or greater than 1 and false discovery rate (FDR) less than 0.05 (Varet et al., 2016). GO and KEGG enrichment analyses of DEGs were further implemented by employing the cluster Profiler R package (version 4.1.3). After 4 months of planting, cassava leaves were collected and three biological replicates were used for all experiments.

### The identification and quantification of anthocyanin content in cassava leaves

The identification and quantification of anthocyanin metabolites were performed at the Wuhan MetWare Biotechnology Co., Ltd. (Wuhan, China). The extraction of samples was analyzed using a UPLC-ESI-MS/MS system (UPLC, ExionLC™ AD, https://sciex.com.cn/; MS, Applied Biosystems 6500 Triple Quadrupole, https://sciex.com.cn/). The analytical conditions of UPLC were as follows: column is ACQUITY BEH C18 (Waters, 1.7 µm, 2.1 mm*100 mm); the solvent system is water (with 0.1% formic acid) and methanol (with 0.1% formic acid) with four gradient programs 95:5 V/V, 50:50 V/V, 5:95 V/V, and 95:5 V/V hold for 10 min, 6 min, 14 min, 14 min, and 2 min, respectively; flow rate is 0.35 mL/min; temperature is 40°C; injection volume is 2 μL. Analyst 1.6.3 software (Sciex) was used for data acquisitions. The MetWare MWDB database was used to identify the metabolites through different parameters such as retention time, m/z value, and fragmentation mode. Differentially accumulated metabolites of filtering conditions were as follows: p-value < 0.05, |log_2_ (FC)| is equal or greater than 1, and variable importance in projection (VIP) is equal or greater than 1. To study the accumulation of specific metabolites, the R package (version 4.1.3) was used to perform principal component analysis (PCA) and orthogonal partial least squares-discriminant analysis (OPLS-DA). After 4 months of planting, cassava leaves were collected and three biological replicates were performed for each experiment.

### qRT-PCR involved in anthocyanin biosynthetic pathway

The total cDNA of cassava leaves were synthesized using the HiScriptIII 1^st^ Strand cDNA Synthesis Kit (Vazyme, Code No. Q312-01). The cDNA diluted to 10-fold by MillQ was used as a template to measure the genes’ relative expression level. The specific primers for genes involved in the anthocyanin biosynthetic pathway and the cassava internal control gene (*actin*) are listed in **Table S1**. qRT-PCR was conducted with a real-time PCR ABI 6800 system using the ChamQ SYBR qPCR Master Mix (Vazyme, Code No. Q311-02). The comparative CT method (2^-ΔΔCT^) was used to quantify the genes’ relative expression (Schmittgen and Livak, 2008). Three biological replicates were performed for each experiment.

### Combined analysis of the transcriptome and metabolome

According to the results of the analysis of the combination of metabolites and transcriptome differential gene detected in this study, the differential genes and all metabolites were mapped into the KEGG pathway map to better understand the relationship between genes and metabolites. Pearson correlation coefficients were calculated to integrate transcriptome and metabolome data. For the joint analysis between the transcriptome and metabolome, the screening criterion was a Pearson correlation coefficient great than 0.8.

### VIGS in cassava

For vector construction, 300 bp *MeANR* was cloned into pCsCMV-NC (negative control) as described by Tuo et al., 2021. The experiment materials used in VIGS were the stems of SC9 which were planted in nutrient-rich soil in an air-conditioned growth room (16-h photoperiod; 2000 lux light intensity) for 20 days. The inoculation method was followed as reported by An et al., 2022. Transformed *Agrobacterium tumefaciens* was precipitated, resuspended, and injected into the cassava leaves and allowed to grow in the greenhouse for 28 dpi. After that, the expression level of the targeted genes in VIGS-*MeANR* silenced lines was detected. Three *MeANR* silencing plants and SC9 were tested. All the primers involved are shown in **Table S1**.

### Statistical analysis

Statistical analysis was performed using Excel 2010 software (Microsoft Office, USA). Data are presented as means ± standard deviations (SD). The levels of statistical significance were analyzed by the least significant difference (Kruskal-Wallis test, *p* < 0.05).

## Results

### The total anthocyanin content in the leaves of different cassava varieties

**Figure 1:**
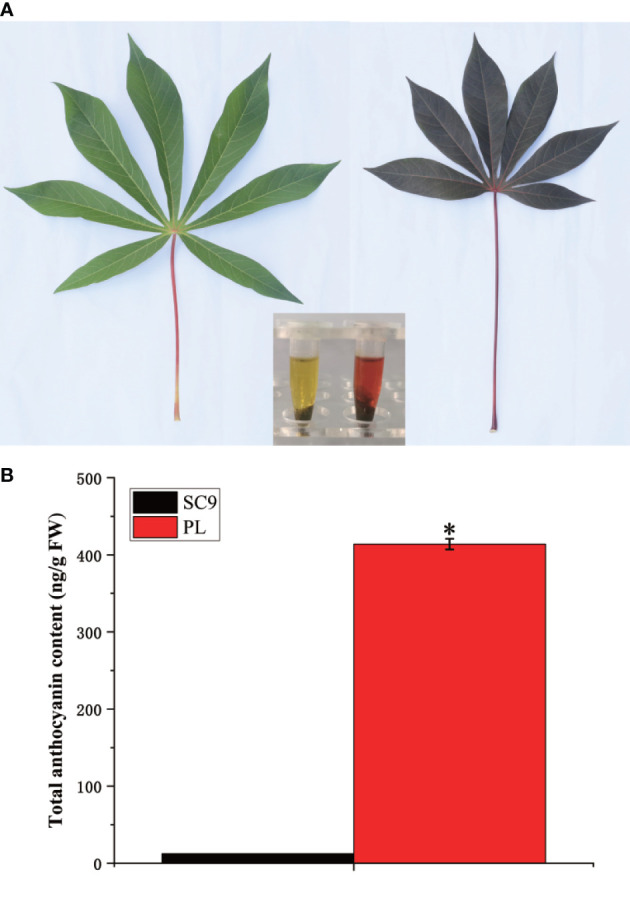
The phenotype and the total anthocyanin content between SC9 and PL are characterized. **(A)** The color of the cassava leaf and extraction between SC9 and PL are shown. Bar: 1 cm. **(B)** The total content of anthocyanin between SC9 and PL was measured. Error bars indicate the standard deviation (SD). Significant differences are indicated by an asterisk (*p < 0.05).

An important pigment, anthocyanin, plays a vital role in the formation of color in cassava leaves. In this study, two different varieties, SC9 and PL, were selected as materials. The content of the total anthocyanins in SC9 and PL was extracted using the methanol-HCl method. The results indicated that the extraction color of SC9 and PL leaves displayed yellow-green and purple-red colors, respectively. The content of total anthocyanins of PL leaves was 413.83 ng/g of fresh weight (FW) which was significantly higher than the total anthocyanins content of SC9 (12.39 ng/g FW) (**Figure 1**) (Kruskal-Wallis test; *p* < 0.05).

### The identification of flavonoids and anthocyanin metabolites in cassava leaves

To investigate the differential flavonoids and anthocyanin metabolites between SC9 and PL, three replications of each sample were analyzed by UPLC/ESI-Q TRAP-MS/MS analysis. A total of 107 flavonoid metabolites were identified in different cassava leaves. PCA analysis indicated that the metabolites of SC9 and PL could be clearly distinguished (**Figure S1**). Among these metabolites of flavonoids, rutin is the most abundant in cassava leaves, followed by kaempferol-3-O-rutinoside and quercetin-3-O-glucoside, which account for 97% of flavonoids in cassava leaves; however, this was not significantly different between SC9 and PL.

**Figure 2:**
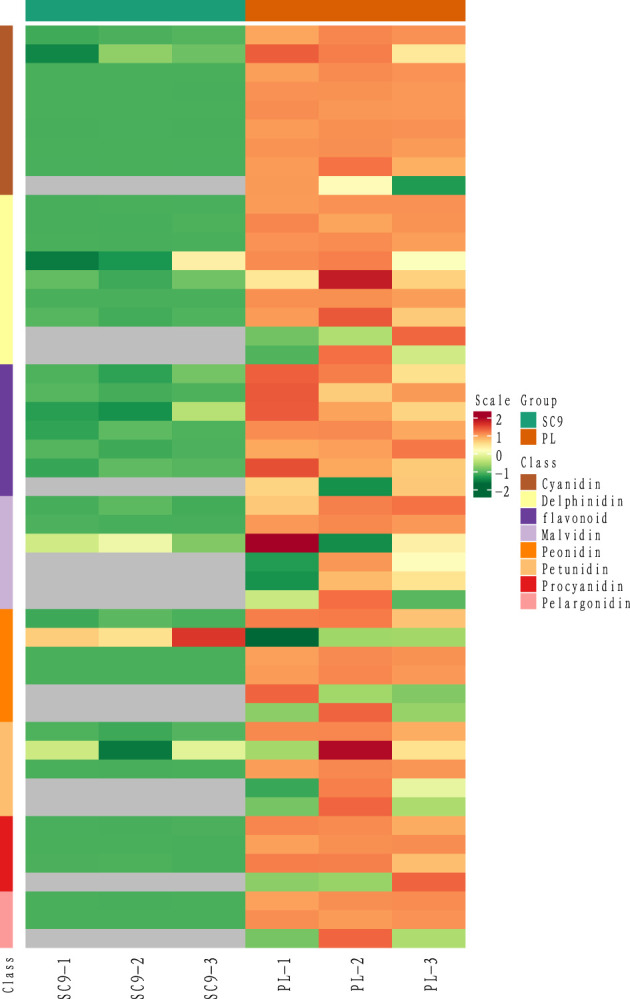
The heatmap of different classes of anthocyanin. The horizontal axis stands for sample processing information, and the vertical axis stands for metabolite information. The cluster tree on the left is the metabolite clustering tree. The scale is the expression quantity obtained after standardized processing; the red color indicates the higher expression quantity and the grey indicates that the expression quantity is N/A. The group includes SC9 and PL.

SDMs were screened using the VIP value > 1 in the OPLS-DA model and FC ≥ 2 or FC ≤ 0.5 as the identification criterion. A total of 25 SDMs in PL were upregulated compared to SC9. These SDMs were generally classified into eight categories, including cyanidins, delphinidins, flavonoids, malvidins, peonidins, petunidins, procyanidins, and pelargonidins. Interestingly, six categories of the SDMs belonging to the anthocyanins were highly accumulated in PL compared with SC9 (**Figure 2**; **Table 1**).

**Table 1:** The contents of differential anthocyanidins between SC9 and PL.

Compounds	Class	The content of differential anthocyanidins in SC9 (ng/g)	The content of differential anthocyanidins in PL (ng/g)	Fold Change
Cyanidin-3-(6-O-p-caffeoyl)-glucoside	Cyanidin	8.625 ± 0.432 b	26.299 ± 0.770 a	3.05
Cyanidin-3,5-O-diglucoside		0.0122 ± 0.001 b	2.610 ± 0.068 a	213.61
Cyanidin-3-O-glucoside		0.065 ± 0.004 b	11.938 ± 0.145 a	182.62
Cyanidin-3-O-sophoroside		0.003 ± 0.000 b	1.870 ± 0.032 a	668.55
Cyanidin-3-O-xyloside		0.006 ± 0.000 b	0.179 ± 0.003 a	31.30
Cyanidin-3-O-rutinoside		0.177 ± 0.004 b	24.937 ± 0.423 a	140.83
Cyanidin-3-O-sambubioside		0.002 ± 0.000 b	0.487 ± 0.041 a	225.77
Delphinidin	Delphinidin	0.159 ± 0.003 b	1.288 ± 0.017 a	8.10
Delphinidin-3-O-glucoside		1.243 ± 0.059 b	9.320 ± 0.337 a	7.50
Delphinidin-3,5-O-diglucoside		0.132 ± 0.004 b	8.728 ± 0.220 a	66.29
Delphinidin-3-O-rutinoside		1.337 ± 0.027 b	295.935 ± 6.207 a	221.33
Delphinidin-3-O-rutinoside-5-O-glucoside		0.168 ± 0.008 b	0.520 ± 0.060 a	3.09
Malvidin-3-O-galactoside	Malvidin	0.004 ± 0.000 b	0.011 ± 0.001 a	2.68
Malvidin-3-O-glucoside		0.011 ± 0.000 b	0.053 ± 0.001 a	4.85
Peonidin-3-O-glucoside	Peonidin	0.002 ± 0.000 b	0.076 ± 0.002 a	166.89
Peonidin-3-O-rutinoside		0.112 ± 0.002 b	15.892 ± 0.303 a	84.94
Petunidin-3-O-glucoside	Petunidin	0.001 ± 0.000 b	0.192 ± 0.006 a	3.14
Petunidin-3-O-rutinoside		0.113 ± 0.003 b	9.615 ± 0.280 a	28.62
Pelargonidin-3-O-glucoside	Pelargonidin	0.087 ± 0.005 b	0.272 ± 0.011 a	34.54
Pelargonidin-3-O-rutinoside		0.126 ± 0.002 b	3.605 ± 0.091 a	141.53

Different letters (a and b) indicate the significant differences determined by the Kruskal-Wallis test (*p* < 0.05, n = 3).

### Differentially expressed genes in cassava leaves

To further understand the anthocyanin biosynthesis in different cassava leaves, transcriptome sequencing was performed. A total of 277,823,272 clean reads were produced by the cassava leaves. The average value of Q20 was 97.46% and the Q30 value was over 92%; the average content of GC was 44.83%, and the sequencing error rates were 0.03%, which was lower than 0.1%. These data indicated that the quality of the sequencing data was good enough for further analysis (**Table S2**).

**Figure 3:**
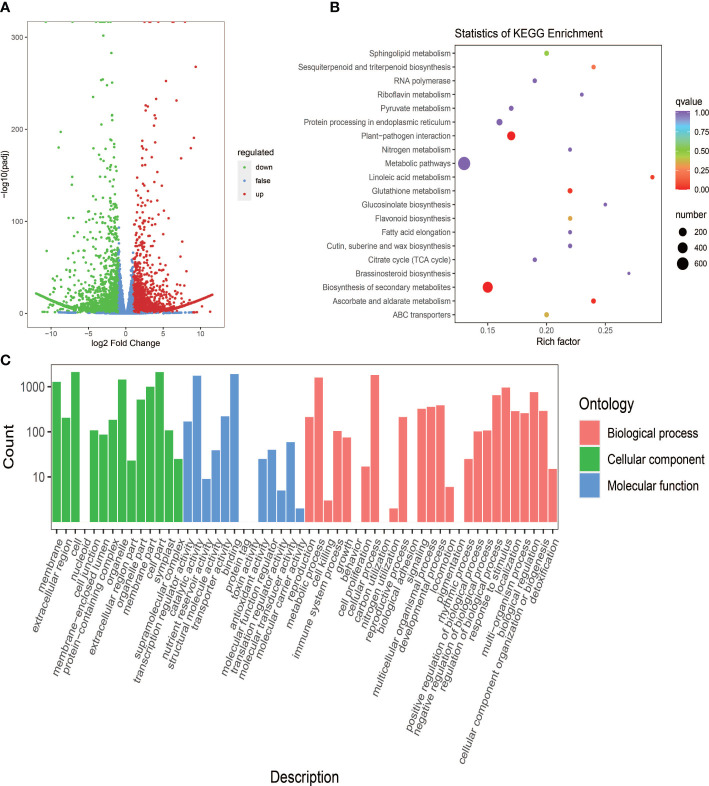
Screening and functional enrichment analysis of DEGs between SC9 and PL. **(A)** The overdistribution of gene expression levels and fold differences between SC9 and PL. The x-axis shows the change in gene multiples, and the y-axis shows the significance level of differentially expressed genes. The red dots stand for up-regulated differential genes, the green dots stand for down-regulated differential genes, and the blue dots stand for non-differentially expressed genes. **(B)** KEGG pathway enrichment of DEGs. The x-axis shows the Rich factor, and the y-axis shows the KEGG pathway. The larger the Rich factor the greater the degree of enrichment. The larger the dot the more the number of enriched differential genes in those pathways. The redder the dot the more significant the enrichment. **(C)** GO enrichment analysis of the 4,846 DEGs in three functional groups (Biological process, Cellular component, and Molecular function) between SC9 and PL. The x-axis shows the secondary entries, and the y-axis shows the number of entry differential genes.

With the filter criteria |Log_2_FC| ≥1 and FDR < 0.05, 4,846 DEGs were identified in the cassava leaves, including 2,349 up-regulated genes and 2,497 down-regulated genes in PL compared to SC9 (**Table S4**). The volcano plots were performed to show the overall distribution of gene expression levels (**Figure 3A**). In order to characterize the function of DEGs, GO and KEGG enrichment analyses were performed. The results showed that these DEGs were associated with biological processes, cellular components, and molecular functions (**Figure 3B**). Combined with the KEGG annotation database, a number of DEGs were enriched in many metabolic pathways, including secondary metabolites biosynthesis and flavonoid biosynthetic pathways (**Figure 3C**). The homologous genes associated with anthocyanin biosynthesis in PL were calculated and compared with SC9 (**Table S3**).

### The combined analysis of the transcriptome and metabolome

**Figure 4:**
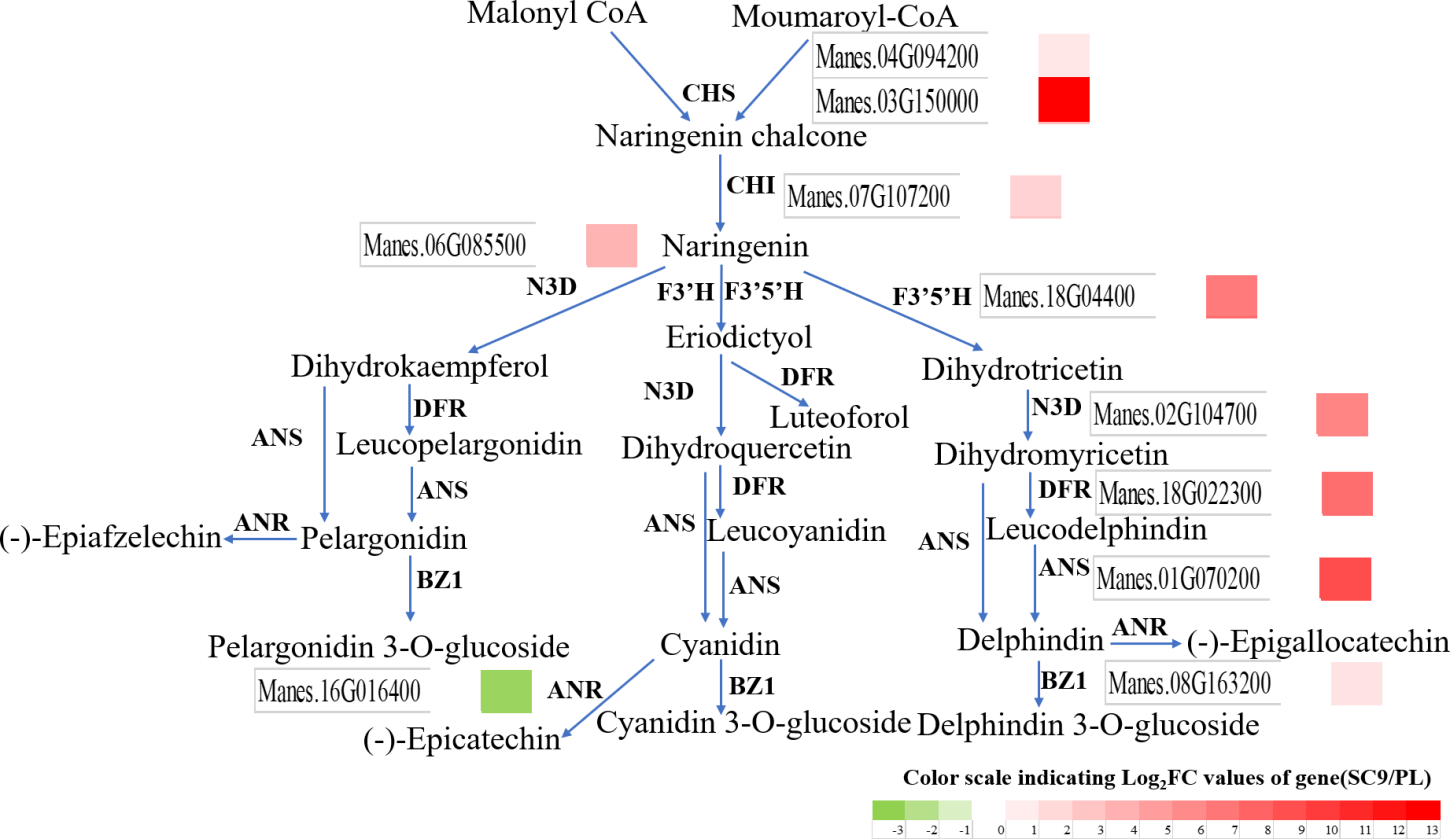
SDMs and DEGs were altered in the anthocyanin biosynthetic pathway between SC9 and PL. Green and red colors indicate the down-regulation and up-regulation of DEGs, respectively.

**Figure 5:**
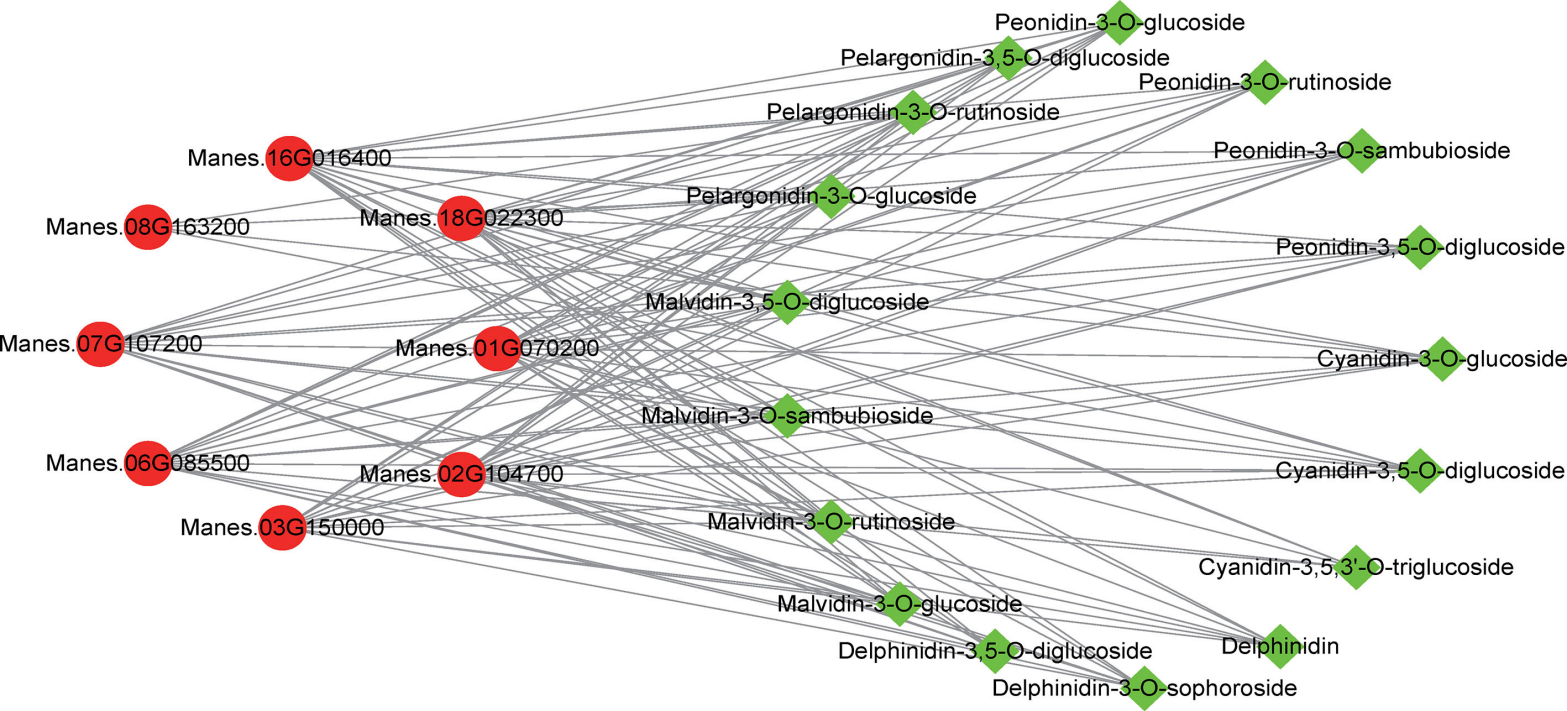
Network analysis of SDMs and DEGs in the anthocyanin biosynthetic pathway between SC9 and PL. Green squares indicate metabolites in the anthocyanin biosynthetic pathway, and red circles indicate genes expressed in the anthocyanin biosynthetic pathway. These SDMs and DEGs were analyzed by the criteria |Pearson’s correlation coefficient| ≥ 0.8.

To further elucidate the relationship between metabolite accumulation and gene expression in cassava leaves, the analysis in combination with transcriptomic and metabolomic data was performed and illustrated. In anthocyanin biosynthesis, 25 SDMs and 10 DEGs were screened and analyzed using correlation analysis after the criteria |Correlation| ≥ 0.8. These results demonstrated that the anthocyanin metabolite changes were significantly associated with the gene expression in the anthocyanin biosynthetic pathway (**Figure 4**). The SDMs such as delphinidin-3-O-sophoroside, malvidin-3-O-sambubioside, and peonidin-3,5-O-diglucoside had a higher correlation with Manes.16G016400 (*MeANR*), while malvidin-3-O-glucoside, delphinidin, and peonidin-3-O-rutinoside had a higher correlation with Manes.01G070200 (*MeANS*) (**Figure 5**). These results indicated that these SDMs and DEGs may participate in anthocyanin biosynthesis.

### Alterations of anthocyanin biosynthesis in PL compared to those in SC9

**Figure 6:**
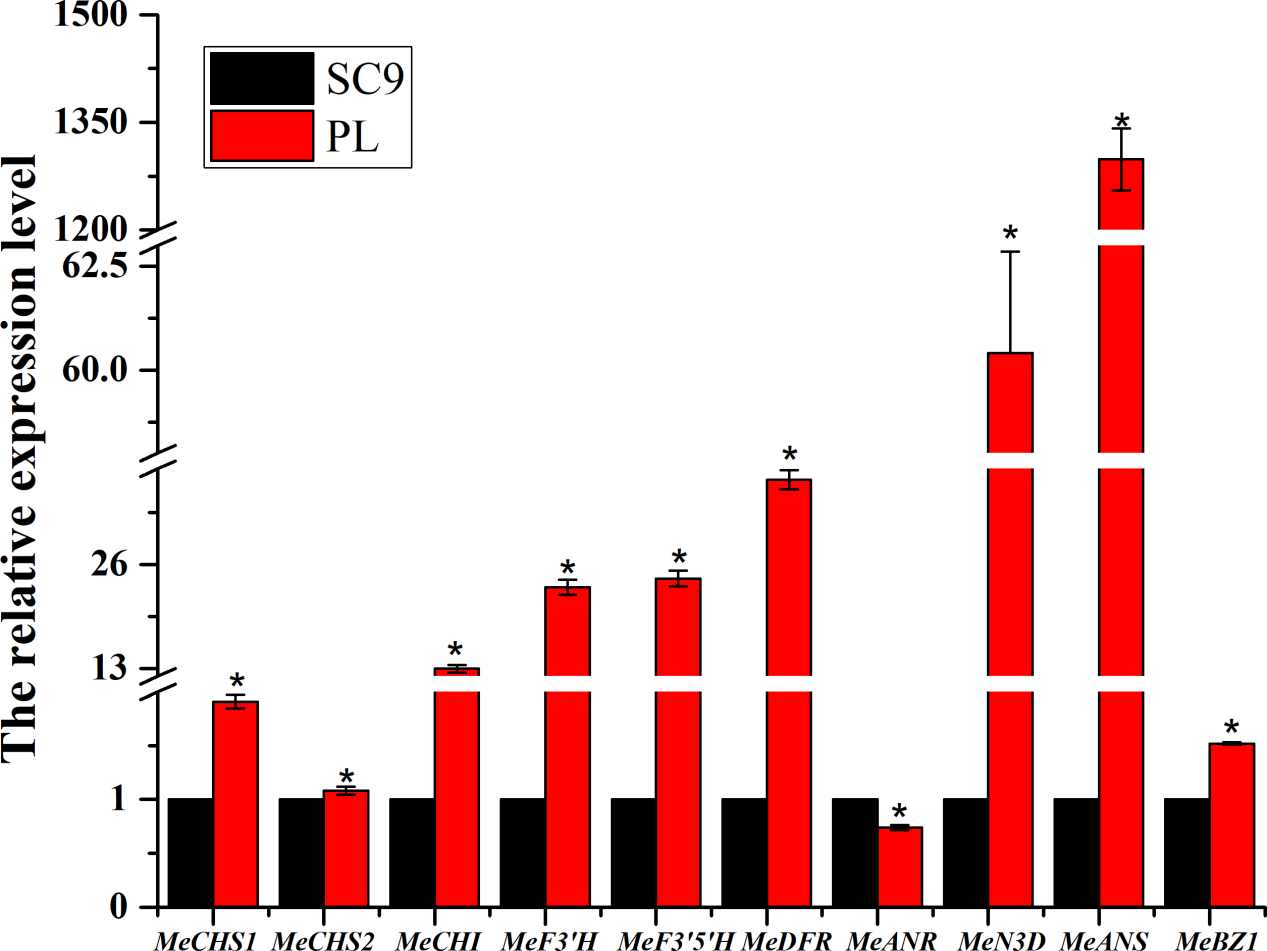
qRT-PCR analysis of the expression of anthocyanin biosynthetic pathway-associated genes. Leaves from cassava plants were used for each RNA extraction (three RNA extractions per plant; n = 3). Data indicate means ± SD. Asterisks indicate a significant reduction or increase compared to the control plants. Significant differences are indicated by an asterisk (*p < 0.05).

The relative expression levels in 10 genes of the anthocyanin biosynthesis including *MeCHS*, *MeCHI*, *MeN3D*, *MeF3’H*, *MeF3’5’H*, *MeDFR*, *MeANS*, and *MeBZ1*, except *MeANR*, were significantly up-regulated in PL than in SC9 leading to high anthocyanin accumulation. To verify the credibility of the transcriptome information, we further selected ten DEGs to validate the sequencing results. The qRT-PCR results were consistent with the RNA-Seq results, suggesting that those DEGs may be involved in anthocyanin biosynthesis (**Figure 6**). The homologous genes associated with anthocyanin biosynthesis were also confirmed by qRT-PCR analysis (**Figure S2**).

### Effects of transcription factors on metabolite accumulation between SC9 and PL leaves

Transcription factors (TFs) regulate the key enzyme expression and participate in secondary metabolism biosynthesis processes in plants. More than 15 TF families including 210 differentially expressed TFs were found in both SC9 and PL leaves in the study (**Table S5**). The most abundant difference expressed transcription factors (DETFs) were MYB-dominant protein (23.24%, 34 were up-regulated and 9 were down-regulated), basic helix-loop-helix protein (bHLH) (15.68%, 15 were upregulated and 14 were downregulated), NAC domain-containing proteins (12.97%, 16 were up-regulated and 8 were down-regulated), followed by AP2/ERF (9.19%, 5 were up-regulated and 12 were down-regulated), WRKY (6.49%, 10 were up-regulated and 2 were down-regulated), Zinc finger (6.49%, 6 were up-regulated and 6 were down-regulated), GARS (5.95%, 5 were up-regulated and 6 were down-regulated), and other TFs (**Table 2**). These TFs may participate in anthocyanin and flavonoid biosynthesis or other secondary metabolism biosynthesis in cassava leaves.

**Table 2:** Transcription factors found between SC9 and PL leaves.

Gene	Enzyme	Numbers of all TFs	Numbers of upregulated	Numbers of downregulated
MYB	MYB TF	43	34	9
bHLH	Basic helix-loop-helix protein	29	15	14
NAC	NAC domain-containing protein	24	16	8
AP2/ERF	Ethylene-responsive TF	17	5	12
WRKY	WRKY DNA-binding protein	12	10	2
Zinc finger	Zinc finger protein	12	6	6
GARS	GRAS domain	11	5	6
MADS	MADS-box TFs	8	7	1
HSF	Heat shock factor protein	7	2	5
B3	B3 domain and auxin response	6	3	3
Trihelix	Trihelix DNA-binding protein	5	4	1
bZIP	Basic leucine zipper	4	1	3
HD-ZIP	Homeodomain zipper protein	4	2	2
SBP	Squamosa promoter-binding protein	4	4	0
GATA	GATA-binding protein	3	1	2
Others		25	8	17

### MeANR is necessary for anthocyanin biosynthesis and color formation in cassava leaves

**Figure 7:**
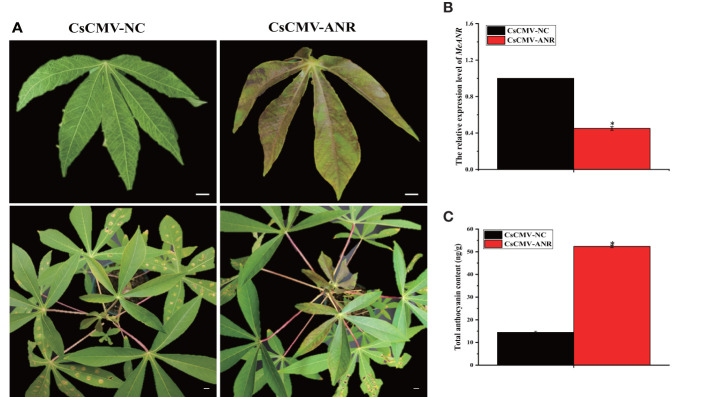
Silencing of *MeANR* in SC9 and its effects on anthocyanin biosynthesis. **(A)** Phenotypes of cassava SC9 leaves and plants displayed by VIGS of *MeANR*. pCsCMV-NC was used as control. The upper photos show a young leaf while the lower photo shows the plant. Bars: 1 cm. **(B)** qRT-PCR analysis on the expression level of *MeANR* in VIGS-*MeANR* plants. Leaves from cassava plants were used for each RNA extraction (three RNA extractions per silencing plant; n = 3). Error bars indicate the standard deviation and significant differences are indicated by asterisk (*p < 0.05). **(C)** The total anthocyanin content was measured in VIGS-*MeANR* plants. Error bars indicate the standard deviation and significant differences are indicated by an asterisk (*p< 0.05).

To investigate whether MeANR was a key enzyme negatively regulating anthocyanin biosynthesis in cassava, VIGS-*MeANR* silenced plants were synthesized and characterized in SC9 after 28 dpi. VIGS-*MeANR* silenced plant presented the altered phenotype of cassava leaves, partially from green to purple color (**Figure 7A**). The transcript level of *MeANR* and the total anthocyanin content were also measured in VIGS-*MeANR* silenced plant. The results showed that the expression level of *MeANR* was reduced by approximately 60% compared with the control plant (**Figure 7B**). The content of the total anthocyanin in the VIGS-*MeANR* silenced plant was 52.46 ng/g, which was higher than that in the control plant (**Figure 7C**). Furthermore, the metabolites in anthocyanin biosynthesis were analyzed, and it was found that these metabolites were highly accumulated in VIGS-*MeANR* silenced plants (**Table 3**). These results indicated that MeANR is a key factor that negatively regulates anthocyanin biosynthesis and color formation in cassava leaves.

**Table 3:** Significantly different metabolites in the anthocyanin biosynthetic pathway of the VIGS-MeANR plant in comparison with the control plant.

Compounds	Class	The content of differential anthocyanidins in SC9 (ng/g)	The content of differential anthocyanidins in PL (ng/g)
Cyanidin-3-O-sambubioside-5-O-glucoside	Cyanidin	0.0027 ± 0.002 b	0.0043 ± 0.0004 a
Cyanidin-3,5,3’-O-triglucoside		0.0062 ± 0.0010 b	0.0105 ± 0.0021 a
Cyanidin-3,5-O-diglucoside		1.2208 ± 0.0297 a	1.3163 ± 0.0773 a
Cyanidin-3-O-glucoside		5.7990 ± 0.0923 b	22.5155 ± 1.0734 a
Delphinidin-3-O-sophoroside	Delphinidin	0.1453 ± 0.0025 b	0.1689 ± 0.0092 a
Delphinidin-3-O-arabinoside		0.0267 ± 0.0015 a	0.0210 ± 0.0013 b
Delphinidin		0.0177 ± 0.0018 a	0.0136 ± 0.006 b
Delphinidin-3,5-O-diglucoside		5.724 ± 0.0672 a	5.9805 ± 0.2967 a
Malvidin-3-O-sambubioside	Malvidin	N/A	0.0134 ± 0.0015
Malvidin-3-O-arabinoside		0.0335 ± 0.0007 a	0.0284 ± 0.0013 b
Malvidin-3-O-rutinoside		0.1724 ± 0.0025 b	0.2028 ± 0.0077 a
Malvidin-3,5-O-diglucoside		0.0203 ± 0.0015 a	0.0155 ± 0.0007 b
Malvidin-3-O-glucoside		0.0104 ± 0.0012 b	0.0180 ± 0.0006 a
Pelargonidin-3,5-O-diglucoside	Pelargonidin	0.0384 ± 0.0018 b	0.0496 ± 0.0027 a
Pelargonidin-3-O-sambubioside-5-O-glucoside		0.0176 ± 0.0003 b	0.0265 ± 0.0043 a
Pelargonidin-3-O-glucoside		0.6877 ± 0.0233 b	0.8622 ± 0.0330 a
Peonidin-3-O-sambubioside	Peonidin	0.0433 ± 0.0062 b	0.0604 ± 0.0040 a
Peonidin-3-O-rutinoside		0.1875 ± 0.0066 b	20.6837 ± 0.0041 a
Peonidin-3,5-O-diglucoside		0.0235 ± 0.0037 a	0.0337 ± 0.0016 b
Peonidin-3-O-glucoside		0.0612 ± 0.0013 b	0.0686 ± 0.0021 a
Petunidin-3-O-arabinoside	Petunidin	0.0626 ± 0.0028 a	0.0553 ± 0.0037 b

Different letters (a and b) in a row indicate significant differences determined by the Kruskal-Wallis test (*p* < 0.05, n = 3).

## Discussion

Anthocyanins are secondary metabolites in plants and have important biological functions in antioxidation, defense regulation, and protection against environmental stresses (Shen et al., 2018; Jiang et al., 2020). In the study, we found that the anthocyanin contents in PL were higher than that in SC9 (**Figure 1**) which was consistent with the finding of the high anthocyanins content in purple leaves of tea cultivar “Zijuan” (Li et al., 2017) and “Baitang” tea (Rothenberg et al., 2019). Interestingly, a total of 20 significantly different anthocyanin metabolites were identified in cassava leaves by UPLC/ESI-QTRAP-MS/MS, which were highly accumulated. Among these anthocyanins, the content of three anthocyanins, i.e., cyanidin-3-(6-O-p-caffeoyl)-glucoside, cyanidin-3-O-rutinoside, and delphinidin-3-O-rutinoside was the most abundant in PL. The cyanidin-3-O-sophoroside content was 668.55-fold higher in PL compared with SC9 and the content of delphinidin-3-O-rutinoside was highest in PL with 295.935 ng/g of fresh weight (**Table 1**). These results suggest that delphinidin-3-O-rutinoside is the major anthocyanin in PL, which may result in the purple pigmentation of cassava leaves.

Anthocyanin biosynthesis was regulated by a series of enzymes. In the study, differentially expressed genes involved in anthocyanin biosynthesis were identified by transcriptome analysis in cassava leaves (**Table S3**). In the anthocyanin biosynthesis, the expression levels of *MeCHS*, *MeCHI*, *MeF3’H*, *MeF3’5’H*, *MeDFR*, *MeANS*, and *MeBZ1* were up-regulated in PL than in SC9 (**Figure 6**), except *MeANR.* ANS is a key enzyme of the anthocyanin pathway that plays an important role in anthocyanidin formation (Ben-Simhon et al., 2015). In our study, the expression level of *MeANS* was the highest among these genes in PL vs. SC9. Some studies also showed that overexpression of *SmANS* or *StANS* increased anthocyanin content in *Salvia miltiorrhiza* and *Solanum tuberosum*, respectively (Li et al., 2019a; Zhang et al., 2020). *ANR* genes (*MrANR1, 2* and *MiANR1-1, 1-2, and 1-3*) are the critical factors in proanthocyanidins (PA) biosynthesis, and there is competition between the anthocyanin and PA biosynthetic pathways (Tan et al., 2018; Li et al., 2019b). Simultaneously, the expression level of *MeANR* was significantly down-regulated in PL. We also used the VIGS system to rapidly analyze the function of *MeANR*. VIGS-*MeANR* silenced plant regulated the altered phenotype of cassava leaves, partially from green to purple color (**Figure 7A**), resulting in a significant increase of the total anthocyanin content (**Figures 7B, C**). The metabolites of anthocyanin were also highly accumulated in VIGS-*MeANR* silenced plants (**Table 3**). The results demonstrated that MeANR is a negative regulator of anthocyanin biosynthesis in cassava. In grape berries, VIGS-*VvANR* silencing increases the expression of *VvANS* and promotes anthocyanin accumulation (Yang et al., 2022). In short, the high expression level of *MeANS* and the low expression level of *MeANR* may promote the accumulation of anthocyanin, leading to the formation of purple leaves. At present, most of the cassava varieties have green leaves. In order to develop the varieties with higher anthocyanin content in leaves, silencing or knock-out *MeANR* could be done in cassava varieties with green leaves so that the varieties with higher anthocyanin leaves might be available and, thereby, fulfill the requirements for food and feeding in the future.

TFs play crucial roles as regulators in anthocyanin biosynthesis. MYB, bHLH, WRKY, WD40, zinc finger, and MADs proteins have been reported for regulating anthocyanin biosynthesis (Terrier et al., 2008; Lloyd et al., 2017; Trainin et al., 2021). To date, the regulation of anthocyanin biosynthesis was known to be not only controlled by a single TF but also regulated by TF/TFs combined with some proteins via an anthocyanin biosynthesis network. A specific example is the MYB-bHLH-WD40 (MBW) ternary complex of transcription factors involving anthocyanin accumulation and inhibition between Arabidopsis, Maize, and Cauliflower (Hichri et al., 2011; Petroni and Tonelli, 2011; Chiu and Li, 2012; Shi et al., 2018). In kiwifruit, an MYB/bHLH complex regulates tissue-specific anthocyanin biosynthesis in the inner pericarp of red-centered kiwifruit *Actinidia chinensis* cv. Hongyang (Wang et al., 2019). Our study found 210 TFs including MYB, bHLH, NACs, AP2/ERFs, WRKY, GARS, Zinc-finger, MADs, HSF, B3, Trihelix, bZIP, HD-ZIP, SBP, and GATA by analyzing the transcriptome data, which showed the significantly different expression levels in PL vs. SC9 (**Table S5**). We speculated that the anthocyanin biosynthesis may be regulated by these transcription factors in cassava and their biological functions need to be further studied.

## Data availability statement

The datasets presented in this study can be found in online repositories. The names of the repository/repositories and accession number(s) can be found below: https://www.ncbi.nlm.nih.gov/PRJNA897109.

## Author contributions

This study was designed by JC and SC. The experiment was performed by XL and FA. Data analysis was finished by JX, WZ, ZW, WO, and KL. Author XL wrote the paper. JC and SC modified the manuscript. All authors reviewed and approved the manuscript.
