# Supplementary figures and images for: Integrative analysis of metabolome and transcriptome reveals the mechanism of color formation in cassava (Manihot esculenta Crantz) leaves
Source: Front Plant Sci. 2023 Jun 9;14:1181257. doi: 10.3389/fpls.2023.1181257 (PMC10289162; doi:10.3389/fpls.2023.1181257)

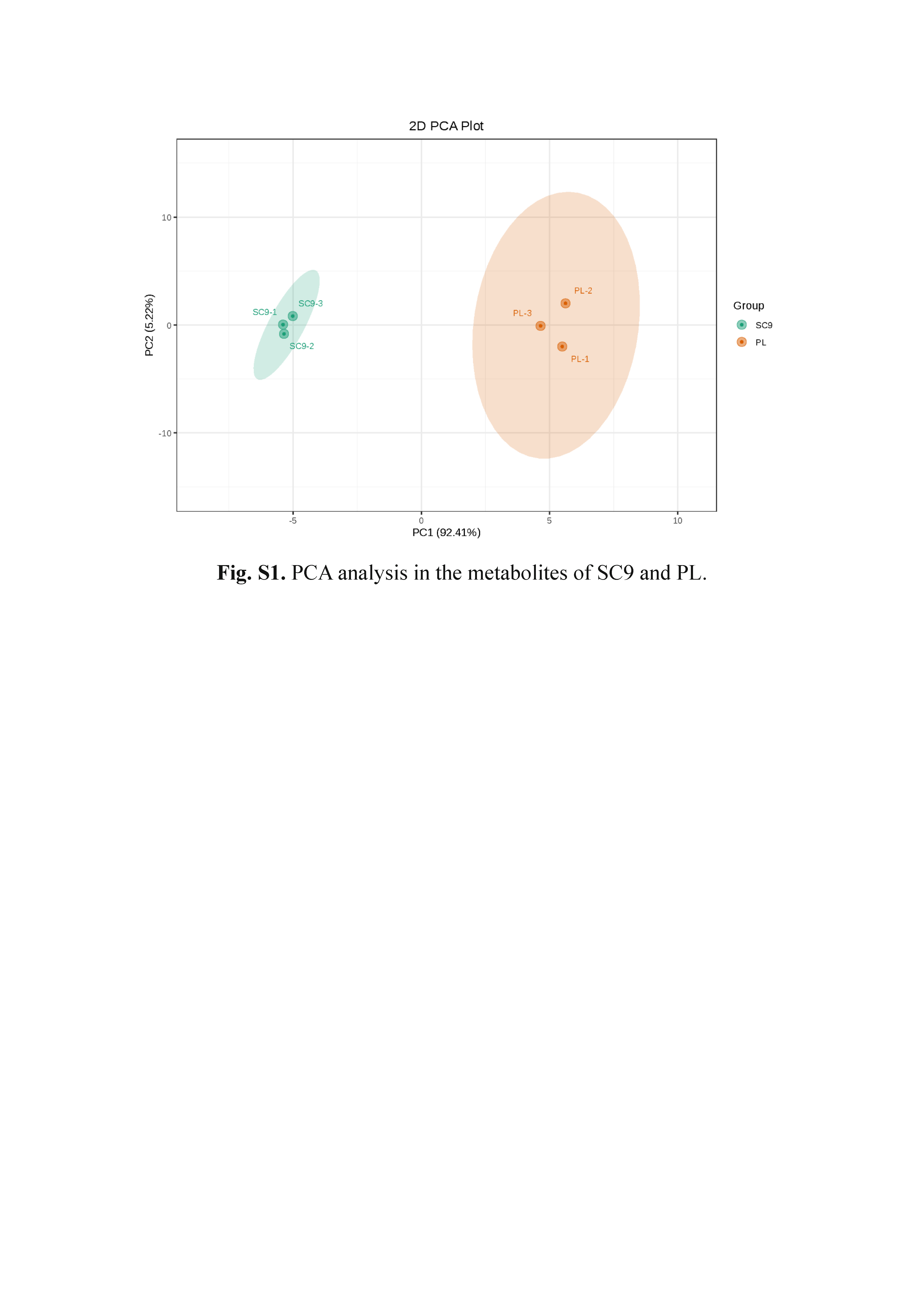

Supplement: Supplementary file 1 [file Image_1.tif]

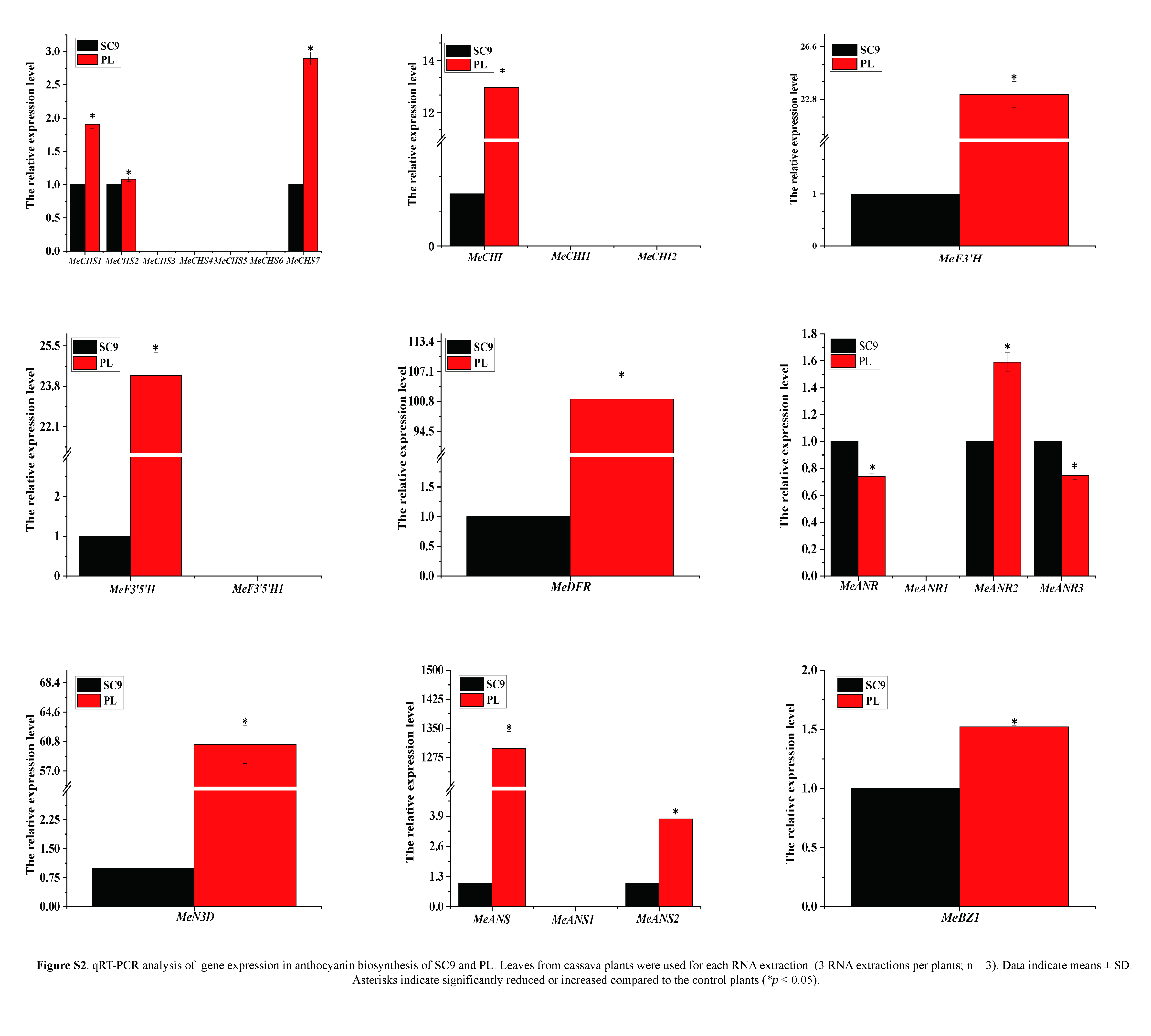

Supplement: Supplementary file 2 [file Image_2.tif]
